# Functional Substrate Mapping in Ablation for Scar-Related Ventricular Tachycardia

**DOI:** 10.31083/RCM45479

**Published:** 2026-02-26

**Authors:** Shiro Nakahara, Hirotsugu Sato, Jason S Bradfield

**Affiliations:** ^1^Department of Cardiology, Dokkyo Medical University Saitama Medical Center, 343-8555 Koshigaya, Japan; ^2^Division of Cardiology, UCLA Health System, Los Angeles, CA 90095, USA

**Keywords:** ventricular arrhythmia, catheter ablation, functional substrate mapping, ventricular tachycardia, multi-electrode mapping, magnetic resonance imaging

## Abstract

Notably, most ventricular tachycardia (VT) episodes in patients with VT attributable to structural heart disease are not hemodynamically tolerated. Therefore, techniques for substrate mapping during stable intrinsic or paced rhythm have been developed that eliminate the need to induce VT. Moreover, advances in catheter technology, enabling high-density multi-electrode mapping of abnormal electrograms, have improved the ability of electrophysiologists to identify the substrate responsible for scar-related VT. In addition to the conventional identification of late potentials and local abnormal ventricular activity (LAVA), several substrate imaging approaches have been developed, including the identification of sites of conduction slowing via isochronal late activation mapping and the modification of wavefronts by changing the pacing site. Further, a new near-field algorithm provides a degree of objectivity to the previously subjective annotations of local potential timing. Additionally, changes in the substrate within the scar, specifically the induction of a line of block and subsequent alteration of a LAVA by decremental conduction, can identify functional abnormal ventricular activity that contributes to the development and maintenance of VT and can further improve the accuracy of substrate mapping. Novel cardiac magnetic resonance imaging and computed tomography analyses, facilitated by specialized software, also provide information for non-invasive estimation of the VT isthmus location. Therefore, continued clinical implementation of these techniques and technologies has the potential to improve safety, reduce the complexity, and expand the number of patients who can safely undergo VT ablation.

## 1. Introduction

**Fig. 1.**
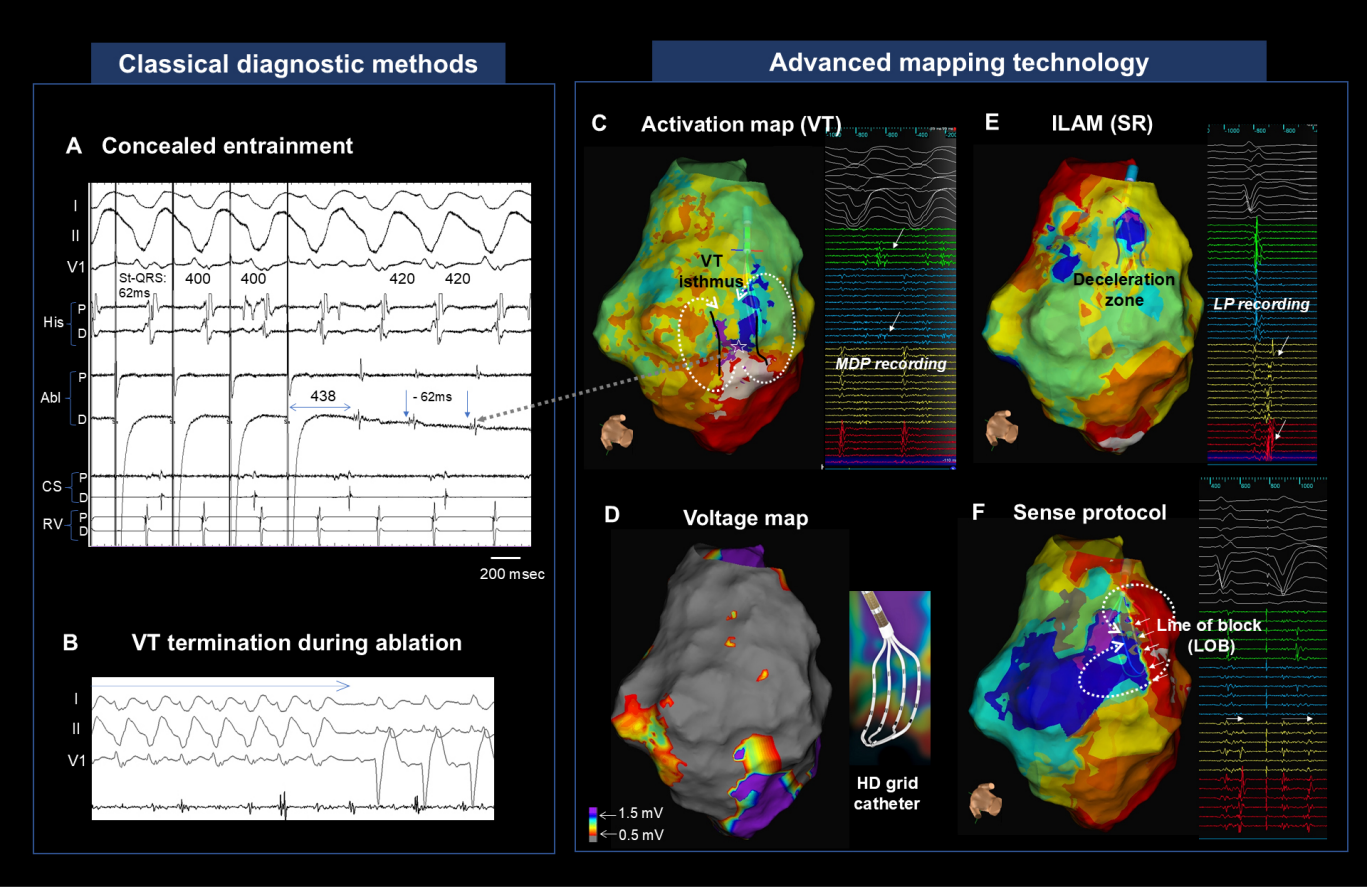
**Identification of critical VT-circuit components using conventional maneuvers and advanced electroanatomic mapping (single case; sequential workflow)**. (A) Post-infarction VT with concealed entrainment. The paced beats show an St–QRS of 62 ms, VT resumes when pacing stops, and the Abl D electrogram precedes QRS onset by 62 ms with near-identical paced/VT morphologies. (B) Radiofrequency ablation at this site terminates VT. (C) VT activation mapping in the same case demonstrates a figure-of-eight reentry with eight sequential isochrones; the termination site in (B) lies within a narrow isthmus (arrow). Right: mid-diastolic potential recorded with an HD Grid catheter. (D) Sinus-rhythm voltage map from the same case. The isthmus area seen in (C) appears uniformly low voltage and is not specifically localizable by voltage alone. (E) Isochronal late activation mapping (ILAM) during sinus rhythm (eight isochrones) shows isochronal crowding defining a deceleration zone (DZ) with adjacent late potentials, spatially close to the termination site in (B)/isthmus in (C). (F) With a Sense-protocol single extrastimulus during sinus rhythm, a line of block aligned with the lateral VT boundary. In the right panel, LP decrement is evident on the extrastimulus beat (second) versus intrinsic rhythm (first), with markers just after the QRS. VT, ventricular tachycardia; St, stimuli; LP, late potential; His, His-bundle electrogram; Abl, ablation catheter; CS, coronary sinus; RV, right ventricle; SR, sinus rhythm.

Electroanatomical mapping-guided catheter ablation plays an important role in reducing episodes of ventricular tachycardia (VT) and potentially avoiding the side effects of antiarrhythmic drugs [1]. Randomized trials and meta-analyses have shown that catheter ablation reduces VT recurrences and implantable cardioverter-defibrillator (ICD) therapies compared with antiarrhythmic drug therapy in patients with structural heart disease, particularly those with ischemic cardiomyopathy and ICDs [2, 3]. In patients with hemodynamically tolerable VT, activation mapping plus entrainment mapping remains the gold standard for identifying critical components of the circuit and successful catheter ablation [4] (Fig. 1A,B). However, inducing VT causes hemodynamic instability in most patients, making mapping during tachycardia difficult. Further, recent reports have shown that many scar-related VTs involve not a two-dimensional (2D), but rather a three-dimensional (3D) circuit [5], and even if the hemodynamics are tolerable, the full extent of the tachycardia circuit can be difficult to elucidate, even with high-density mapping of the endocardium and epicardium. Therefore, multiple substrate-based ablation strategies that estimate the location of critical parts of the VT circuit from electrophysiologic features have emerged as important alternatives to induction and mapping of sustained VT, expanding the population of patients who may benefit from VT ablation [6]. The substrate ablation strategies developed to date include scar homogenization, scar dechanneling, and targeting local abnormal ventricular activity (LAVA) and late potentials (LPs) [7, 8, 9, 10, 11]. However, outcomes of these strategies are still not optimal, with success rate of 70% and procedure-related complications still reported at 5% to 10% [12]. Although reported complications are primarily related to the ablation procedure rather than to the mapping strategy, the potential contribution of prolonged procedures and extensive lesion sets cannot be excluded. Despite the techniques’ utility, procedures targeting all abnormal potentials or scar border zones may necessitate extensive endocardial or epicardial ablation and thereby undesirably prolong procedure time.

In recent years, increasing attention has been given to approaches that detect functional electrophysiologic features within the scar tissue during intrinsic or paced rhythm in patients with scar-related VT. Dynamic changes uncovered may reflect essential aspects of the VT substrate, especially in VT due to a fixed or functional barrier with rate-dependent and variable conductive properties. The goal of these techniques in combination with advanced imaging is to localize the key components of the VT substrate and carry out catheter ablation in a hemodynamically stable rhythm. This review summarizes the latest functional substrate mapping methods along with their key underlying concepts and explores future directions.

## 2. Multi-Electrode Mapping

The basic hypothesis of substrate mapping of myocardial scar-related VT is that a proxy for the VT isthmus can be identified during a stable cardiac rhythm. Surrogates include electrograms (EGMs) with electrical discontinuities such as fragmented potentials, LAVA, and LPs, which are recognized as abnormal ventricular EGMs at sites of slowed activation [9]. Reduction in the size and spacing of the electrodes of mapping catheters has contributed significantly to the characterization of arrhythmic substrates [13]. Multipolar catheters (PENTARAY [Biosense Webster, Irvine, CA]/OCTARAY [Biosense Webster]/OPTRELL [Biosense Webster]/HD Grid [Abbott, Minneapolis, MN]/Orion [Boston Scientific Marlborough, MA]) are advantageous in that they provide (1) high-density mapping and substrate clarity; (2) maximized detection of LAVA and voltage amplitude channels; and (3) provide high accuracy in identifying and delineating LAVA or LPs, while distinguishing near-field components from far-field potentials [14, 15, 16]. VT, in patients with structural heart disease, as described above, is often not hemodynamically tolerated and can have multiple morphologies during the procedure, often preventing acquisition of an activation map. Multi-electrode catheters were developed to allow the atrial and ventricular arrhythmias to be mapped within a reasonable time frame. In addition, automated mapping algorithms have increased the mapping speed and density, facilitating accurate assessment of the location of the VT isthmus. Functional substrate mapping also benefits from a multipolar catheter and can refine the substrate approach and improve sensitivity and specificity of mapping.

The spatial fidelity of EGM analysis depends on electrode size and interelectrode spacing. Smaller electrodes with closer spacing increase near-field resolution and attenuate far-field averaging, thereby improving the detection of fragmented EGMs, LAVA, and LPs [13, 14, 16]. These features also enhance the precision of local activation timing used for functional mapping (e.g., isochronal late activation mapping (ILAM)) [17, 18]. In practice, high-density sampling with multipolar catheters refines substrate characterization within scarred myocardium and sharpens delineation of conduction channels and deceleration zones during stable rhythm [19, 20]. Furthermore, analysis frameworks designed to mitigate the directional dependence of bipolar recordings (e.g., omnipolar vectors and automated near-field algorithms) complement the benefits of closer spacing by improving local activation assignment within low-voltage regions [21, 22]. Examples of functional substrate mapping and VT activation mapping performed with a multipolar mapping catheter are shown in Fig. 1. In a single representative case, we illustrate a step-wise workflow in which conventional activation/entrainment mapping constrains the VT isthmus, while sinus-rhythm functional mapping (ILAM and a Sense-protocol extrastimulus) refines and functionally validates the substrate target under hemodynamically stable conditions. In contrast, voltage mapping alone offers limited specificity for isthmus localization in scarred myocardium—low-voltage areas are often confluent and indistinguishable from the critical channel— as shown in Fig. 1D.

## 3. Isochronal Late Activation Mapping (ILAM)

**Fig. 2.**
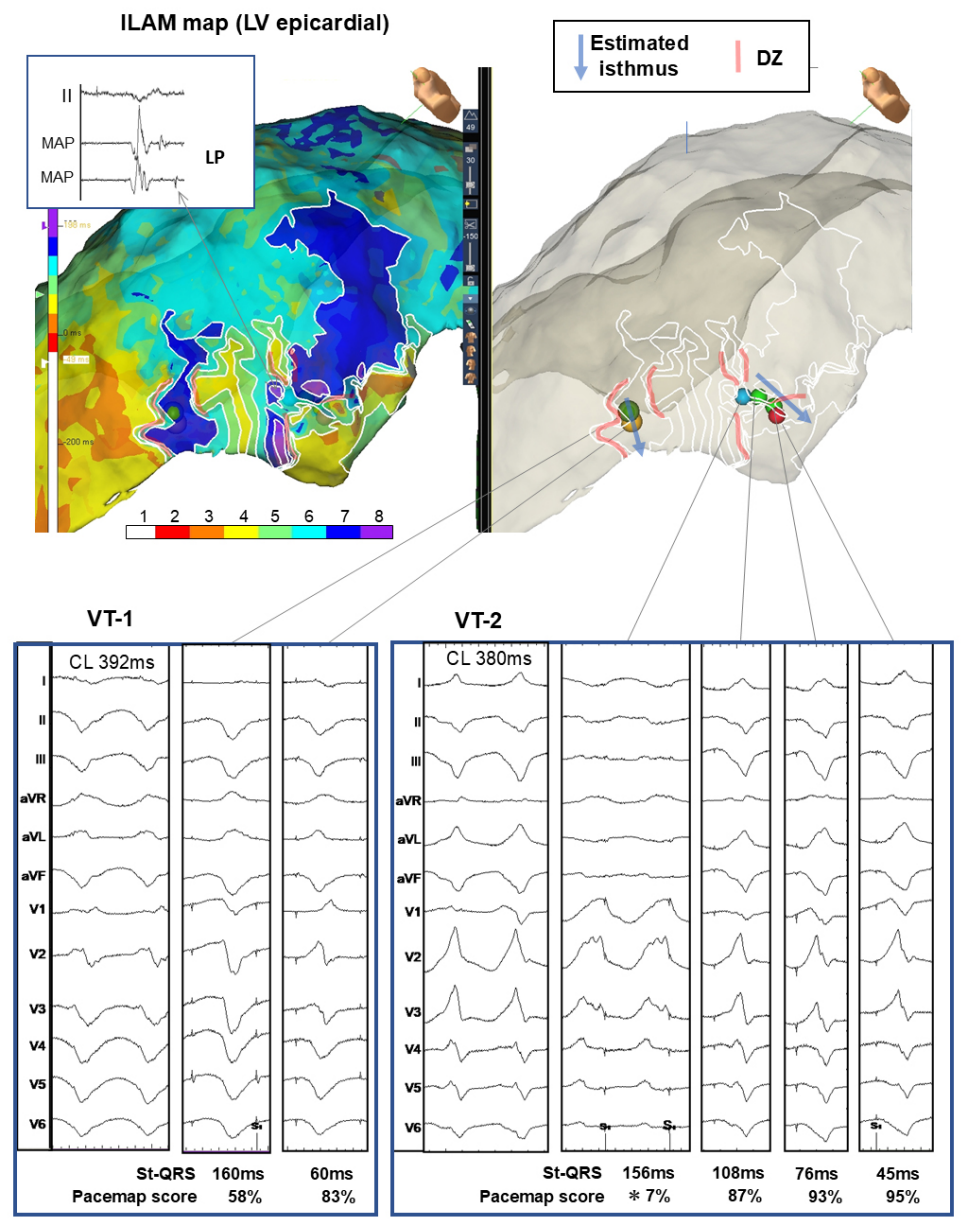
**Isochronal late activation mapping (ILAM) and pace mapping findings in a case of dilated cardiomyopathy with epicardial ventricular tachycardia**. Upper left: Left ventricle (LV) isochronal late activation map obtained in a case of dilated cardiomyopathy with VT originating from the epicardium. A late potential (LP) is seen around the narrow deceleration zone (DZ) in the color band. Top right and bottom: The pace map findings and stimulus-QRS time (latency) for the target VT are shown. The estimated isthmus of the VT (light blue arrow) can be seen around the DZ. Notably, pace mapping in the proximal region of the estimated VT-2 isthmus shows a sudden decrease in the pacemap score (from 87% to 7%) associated with St-QRS prolongation (156 ms), which is thought to indicate a functional substrate (asterisk).

Functional substrate mapping incorporating analysis of multiple wavefronts is gaining attention. This methodology can be used to identify areas where conduction is interrupted or slowed during intrinsic rhythm and areas within the scar tissue that are particularly prone to re-entry. The methodology is expected to refine the substrate-based approach to VT ablation and improve the sensitivity and specificity of mapping in identifying the critical isthmus [19, 20]. Irie *et al*. [23] reported the relation between sinus rhythm late activation zones and critical sites for VT. They described the propagation of ventricular excitation over eight equally distributed activation isochrones (with 12.5% of the ventricular activation comprising each isochrone) in 47 patients with scar-related VT. Critical sites related to the VT isthmuses were often identified outside the slowest isochrone of ventricular activation. Rather, they were identified at more proximal sites, where conduction slows down. Recently, several groups of investigators have reported that zones of slowed or steeply activated conduction time gradients, i.e., conduction delay regions, correspond well to the isthmus of the VT [19, 24, 25] (See Fig. 1E and Fig. 2 for typical isochronal late activation maps). Aziz *et al*. [19] used high-resolution mapping with a multi-electrode catheter to produce an isochronal late activation map of increased density and accuracy in 125 cases. They found that the region of wavefront deceleration visualized by discontinuity or dense clustering of activation colors, ‘isochronal crowding’, most likely included the VT isthmus region. They defined deceleration zones (DZs) as areas 1 cm in radius containing three or more isochrones, and the ensuing DZ-targeted ablation provided good clinical results. Note that there is no clear cut-off value for conduction velocity, as the functional substrate can change dynamically in response to the wave direction and additional stimulation [20]. Furthermore, if no clear functional substrate is identified on activation mapping during sinus rhythm, change in the activation waveform attributable to pacing should be considered. Additional mapping techniques such as pace mapping, entrainment mapping, and limited VT activation mapping at conduction delay sites may help to clarify the area of interest. In recent years, localized wavefront deceleration has also been visualized by means of omnipolar mapping with a multi-electrode grid catheter. This relatively new method of EGM analysis is designed to overcome the “bipolar blindness” that can occur under conventional electroanatomical mapping and to complement the conventional ILAM method, which targets low voltage areas associated with the VT [21].

## 4. Pace Mapping to Identify Critical VT Isthmuses

Pace mapping is a supportive methodology for VT circuit identification [26]. It can be used to identify the presumed exit or isthmus site of the VT circuit, but it is not specific or sensitive enough to be the sole guide for ablation. Automated pace-map matching is now available on several lab systems and 3D mapping systems. The higher the agreement between the ECG morphology during pacing and the tachycardia, the closer the catheter tip to the exit zone of the VT isthmus. Pace-map matching can also detect slow conduction, as indicated by an interval of >40 ms between the pace mapping stimuli and the QRS complex (St-QRS). The site of St-QRS delay is usually within the scar region, as identified by the EGM amplitude, and is likely to be indicative of an isthmus adjacent to the area of conduction block. The St-QRS interval theoretically increases as the pacing site moves from the exit region to the entrance site within the VT isthmus [21]. De Chillou *et al*. [27] reported good correlation between the pace map and the 12-lead ECG recorded during VT at sites close to the isthmus exit but poor correlation between the pace map and the 12-lead ECG recorded during VT at sites close to the isthmus entrance. They reported that an abrupt change in the pace map score identifies the core of the critical isthmus in scar-related VT. Abrupt QRS morphology changes occur as the mapping catheter moves from one side of the mid-isthmus line to the other. Suppose pacing is performed slightly to the side (entry area) of the mid-isthmus during sinus rhythm. In that case, the activation wavefront will propagate in both directions relative to the direction of the isthmus, and that is thought to be a functional response that causes a sudden change in the score. Pace mapping in conjunction with ILAM-based identification of DZs, as described above, is widely used in clinical practice. Conventional pace mapping is primarily used for focal premature ventricular contraction (PVC) ablation; in scar-related reentrant VT, it is typically applied selectively and in combination with substrate-based and functional mapping rather than as a stand-alone strategy. Example output of pace mapping guided by ILAM-identification of the DZ is shown in Fig. 2.

## 5. Alternative Activation Wavefront Analysis

Tung *et al*. [28] were among the early investigators to demonstrate that the direction of ventricular activation wavefronts can significantly influence the electroanatomic characterization of scar tissue during substrate mapping for VT. In their study, voltage maps acquired using multiple wavefronts—achieved through pacing from different sites—revealed marked variability in the extent and distribution of low-voltage areas. Importantly, certain VT-critical sites were not identifiable when mapping was performed using a single wavefront, but became evident when alternative activation vectors were employed. These findings underscore the dynamic nature of functional substrate and the inherent limitations of single-wavefront mapping strategies. Building on this concept, Martin *et al*. [29] performed pacing from three directions (atrial and right and left ventricular pacing) in 22 patients with ischemic cardiomyopathy. They reported that slowing/blocking of the conduction due to changes in the wavefront helped to locate the critical isthmus sites of non-mappable VTs. In particular, the line of block (LOB) and LAVA potentials were found to occur most often on wavefronts perpendicular, rather than parallel, to the VT isthmus (see Fig. 1F). Anter *et al*. [20] also found more ventricular conduction delay sites within the scarred region, similar to the DZs detected by ILAM, when pacing from three directions. Further, treatment outcomes improved by applying radiofrequency ablation to the cumulative area of delayed activation (the total area where the activation time per 10 mm was ≥40 ms). These reported findings indicate that in patients with little or no evidence of localized abnormal ventricular activity during substrate mapping, use of an alternative wavefront of excitation—one perpendicular to the VT isthmus—may improve sensitivity in detecting the substrate and critical sites of re-entry underlying the arrhythmia.

## 6. Frequency Analysis

The ILAM-based approach described above overcomes some problems in defining ‘local’ activation in abnormal EGMs, multicomponent EGMs, split potentials, LAVA, and other features within low-voltage regions by annotating the offset of the last local bipolar EGM. However, signal noise and artefacts may necessitate manual adjustment of the sensitivity and position of the annotation to obtain an accurate isochronal late activation map [17]. Such manual re-annotation can be time-consuming in the era of multi-electrode mapping, where electroanatomical maps can number in the thousands.

**Fig. 3.**
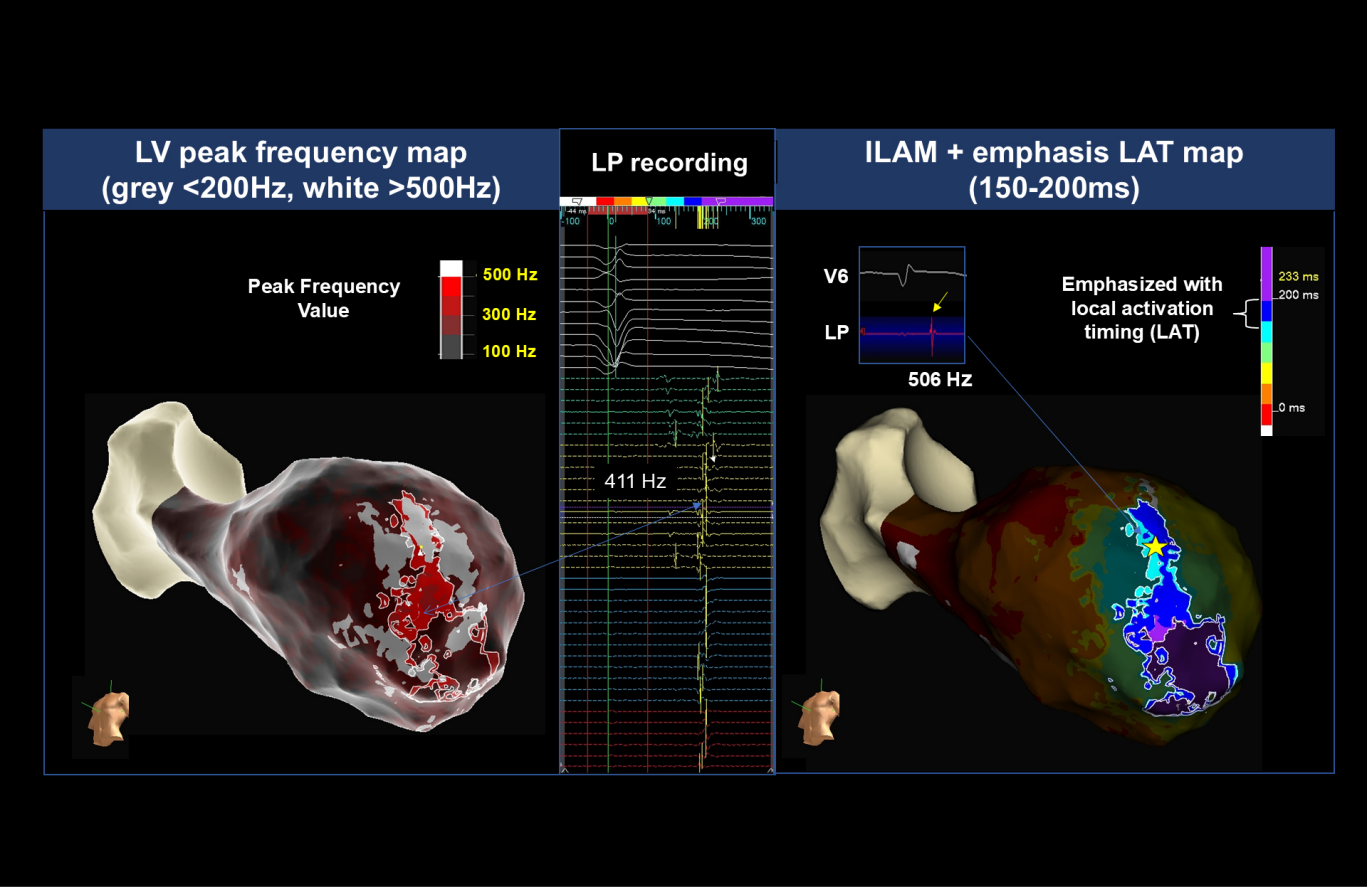
**Peak frequency mapping and isochronal late activation mapping (ILAM) for cases of ischemic cardiomyopathy**. Left panel: example of a left ventricle (LV) peak frequency map obtained from a patient with an extensive chronic anterior myocardial infarct and aneurysmal findings (with adjusted thresholds of EnSite X; grey <200 Hz, white >500 Hz). The automated near-field detection allows rapid identification of the ventricular tachycardia isthmus segments in the near field of the mapped surface without manual re-annotation. Middle panel: Late potential (LP) recording with use of the HD Grid. The LP components are automatically annotated by the near-field algorithm. The arrowed electrogram indicates a relatively high frequency region of 411 Hz at the auto-annotated LP site. Right panel: Isochronal late activation map generated by using the near-field algorithm. The LP upstream timing (local activation timing set to 150 to 200 ms after the R-wave peak) is highlighted as the optimal ablation area for LP elimination (dechanneling approach). This corresponds to the brighter white area in the left image. A region of relatively high frequency (506 Hz) is noted at the auto-annotated LP site (yellow dart). Each LP was eliminated by radiofrequency ablation in the LP area (506 Hz), as indicated by the yellow star.

Furthermore, it is not always clear whether the slowest component of the bipolar potential represents a local (near-field) activation potential or a far-field potential. Therefore, new algorithms are needed to improve the accuracy of real-time annotation of local excitatory activation. Payne *et al*. [18] reported use of a new annotation algorithm of the Abbott EnSiteX electroanatomical mapping system (EnSite OT Near Field) to generate substrate maps for VT ablation. They performed wavelet transform time-frequency analysis using the software bundled within the EnSite system in patients with VT. Their resulting data suggested that the new algorithm could better identify and annotate the region of the intracardiac EGM containing true near-field signals by identifying the timing of the segment of the ECG containing the highest peak frequency (PF). From a retrospective analysis of 25 cases, they found that the potential VT substrate areas, characterized as LPs and LAVAs within low-voltage areas, had PF values higher than those of normal myocardial tissue and scar areas without LPs or LAVAs. Using their PF data, they calculated an optimal PF cut-off of >220 Hz for identifying LP and LAVA EGMs with high sensitivity (91%) and specificity (85%). In addition, in 90% of 10 prospectively enrolled patients, the high PF zone in the low-voltage area was spatially concordant with the ILAM-identified DZ. Areas with specific high-frequency bands can be color-coded on the 3D map, providing excellent visibility of the target area (Fig. 3). In depth details on how the algorithm works have not yet been released, but in general, the algorithm seems to be based on wavelet analysis, and the data can be analyzed in real time during the procedure and thus overcome the problem of short-time Fourier transform, which is suitable for offline analysis [30]. While the PF is useful for displaying the near-field EGMs, in cases where a myocardial isthmus is in the middle layer, the far-field component is likely to be increased and may become a problem [22]. Prospective studies of the usefulness of the algorithm in large numbers of patients are needed. In addition, different PF cut-off values may be required for different pathological conditions and rhythms, such as sinus rhythm and tachycardia.

## 7. Functional Extra-Stimulus Mapping and Beyond

**Fig. 4.**
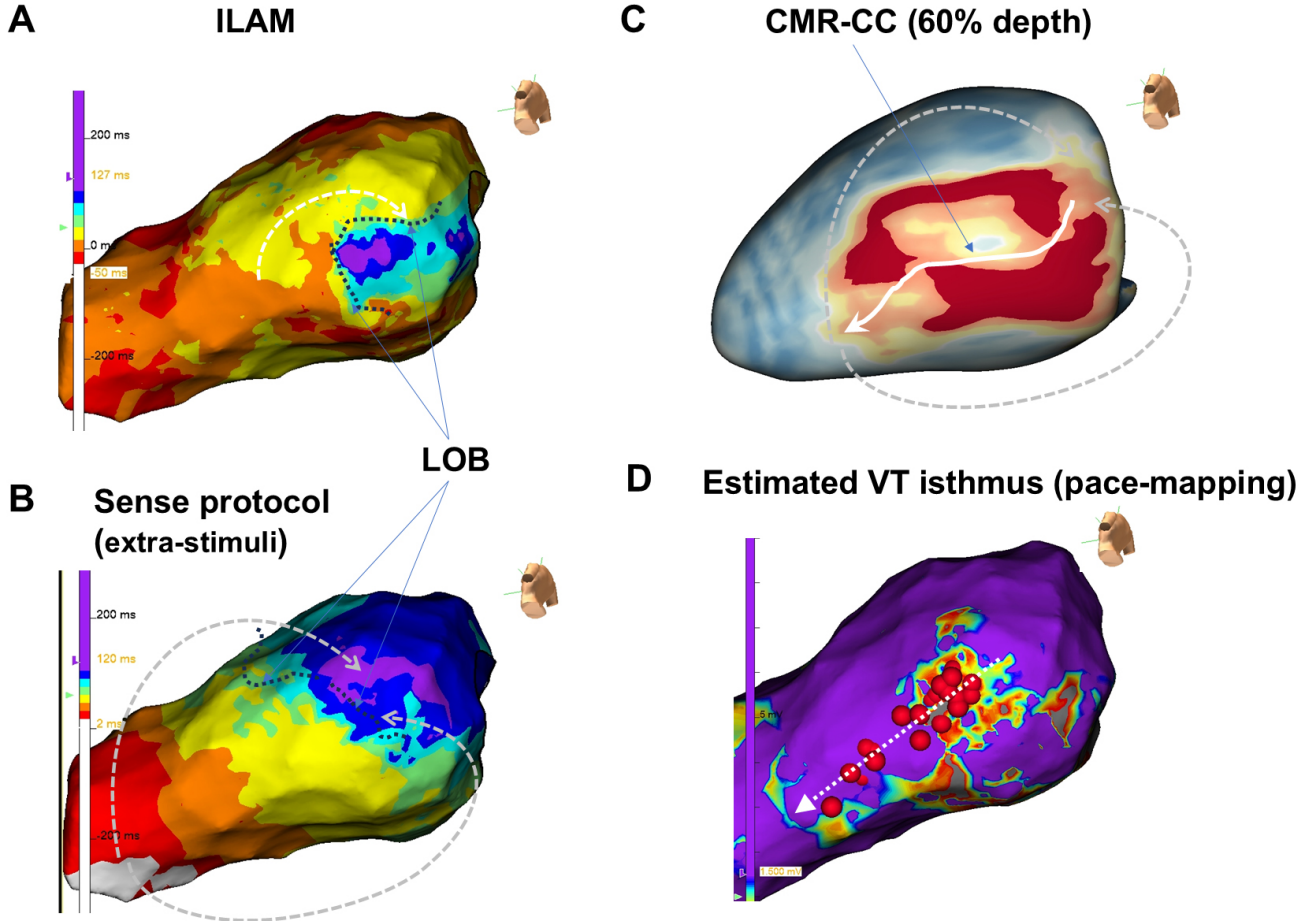
**Comparison of a late gadolinium enhancement cardiac magnetic resonance (LGE-CMR) image and functional map obtained from a patient with ventricular tachycardia (VT) due to ischemic cardiomyopathy**. As the target VT was hemodynamically unstable, a substrate approach was attempted by using LGE-CMR. (A) The isochronal late activation mapping (ILAM) display obtained during sinus rhythm is shown. A U-shaped line of block (LOB) was observed around the deceleration zone in the pre-phase region of the purple phase region on the posterior wall of the basal part of the left ventricle. (B) ILAM display obtained by using the Sense protocol. With early stimulation from the right ventricular apex, the LOB straightened, and the ILAM excitation pattern changed to a boomerang-like pattern. (C) An estimated CMR conduction channel (CMR-CC) was analyzed with use of LGE-CMR and ADAS analysis software. The CMR-CC was observed at a depth of approximately 60% from the endocardium (middle layer of the myocardium). (D) Pace mapping was performed with the CMR-CC used as an index. The estimated channel (dashed arrow) with a good score was identified, and VT could no longer be induced following ablation targeting the channel region.

Difficulties are often encountered in creating isochronal late activation maps and in identifying abnormal local ventricular EGMs. In particular, pathological near-field signals may be obscured by the far-field signals during the intrinsic rhythm [31]. Furthermore, the interpretation of local abnormal EGMs is highly subjective, and DZs identified by ILAM may appear during the main ventricular activity rather than during the late stage of ventricular excitation propagation. To improve the identification of VT-related substrates, an approach using one or more ventricular extrastimuli relative to the baseline rhythm, aimed at increasing the specificity of VT isthmus detection, has been proposed [32]. Antecedent to the functional mapping that incorporates dynamic elements into the static basic EGM rhythm, Jaïs *et al*. [33] used ventricular extrastimulation to identify near-field potentials and sharp high-frequency ventricular potentials in identifying LAVA potentials. Further, Jackson *et al*. [34] inserted a 112-electrode balloon catheter into the left ventricle in six patients undergoing coronary bypass surgery and defined ventricular EGMs showing slow conduction with a decremental extrastimulus as decrement-evoked potentials (DEEPs). They found that DEEPs are spatially consistent with the VT isthmus, and they used a mathematical model to show that the DEEP mechanism is involved in zig-zag conduction within the scar. This approach was refined in a multicenter study and used in a non-surgical, conventional catheter ablation procedure [35]. The multicenter study showed that the DEEP region is more localized in the diastolic pathway of the VT than LPs, and targeting the DEEP region led to favorable clinical outcomes. Acosta *et al*. [32] applied double ventricular extrastimuli and defined the hidden slow conduction EGMs (HSC-EGMs) as the area showing a DEEP-like excitation pattern. HSC-EGMs were found not only in scar border zones; 28.8% were found in areas labeled as normal voltage tissue. Signal intensity maps obtained from contrast-enhanced cardiovascular magnetic resonance (CMR) also showed that HSC-EGMs were located within the scar region. Eventually, targeted ablation of identified HSC sites was associated with a shortened RF energy delivery time during substrate ablation and a decreased incidence of VT induction. Srinivasan *et al*. [36] produced two substrate maps—one by using a multipolar catheter during sinus rhythm and the other by using right ventricular Sense protocol mapping to better identify areas of conduction delay. The protocol involves finding the effective refractory period (ERP) of a single-paced right ventricular (RV) sensed extra beat (without a drive train), delivering single sensed extrabeats at 20 ms above the RV ERP every fifth beat. Further, the method is used to create a template in the form of the 12 surface-based leads of this early stimulation, collect points that match the template, and use the turbo map function to create a substrate map of this type of beat. Eventually, ablation targeting LPs and LAVAs, which were clearly demonstrated by the Sense protocol mapping, did not induce VT in 29 of 30 patients cases, and the VT was resolved in 90%. In recent years, there have been attempts to actively explore DEEPs by performing S3 stimulation (double extrastimuli) as premature ventricular stimulation [37]. However, the unique feature of the Sense protocol is that, unlike DEEP pacing or the S3 protocol, the hemodynamic burden placed on the patient seems to be reduced because there is less additional pacing of the ventricles. Typical output of Sense protocol-based ILAM mapping in patients with ischemic cardiomyopathy is shown in Fig. 1F and Fig. 4B.

Functional extra-stimulus mapping uses short-coupled extrastimuli to unmask DEEPs and related slow-conduction markers that colocalize with the VT isthmus, improving target specificity under sinus rhythm [32, 33, 34, 35, 36, 37]. Early feasibility and multicenter data support clinical utility, particularly with Sense-protocol single extrabeats; however, annotation dependence and center-specific protocols remain, and standardized workflows and prospective validation are needed.

### 7.1 Intramural Delay Mapping

When endocardial and epicardial voltage are confluent and nonspecific, intramural (in-wall) slow conduction can be inferred by high-density orthogonal sampling and endo–epi integration, often revealing a narrow band of fragmented or long-duration signals that bridges otherwise discontinuous surface findings. Such patterns, consistent with three-dimensional reentry, complement ILAM/DEEP when voltage alone lacks specificity [38].

### 7.2 Ventricular Electrogram Duration Map (VEDUM) 

The VEDUM is a functional substrate-mapping technique based on spatial quantification of bipolar electrogram duration to identify compact regions of slow and inhomogeneous conduction. VEDUM was initially introduced during sustained VT to localize zones with markedly prolonged electrogram duration at critical circuit sites, showing close concordance with VT isthmuses and acute termination targets (VEDUM pilot study) [39]. Subsequently, a stable-rhythm implementation was developed and is now the predominant clinical approach: VEDUM areas are delineated during sinus or paced rhythm without programmed extrastimuli, using high-density sampling to reveal slow-conduction corridors and functional lines of block within scar (VEDUM Project Study) [40]. Given potential influences of rhythm context, pacing site/output, mapping density, and positivity thresholds, protocol standardization and external validation remain necessary. Importantly, the recent multicenter VEDUM FREEDOM Study in ischemic cardiomyopathy demonstrated that greater ablation coverage of the VEDUM area was independently associated with a lower risk of VT recurrence during follow-up, supporting a potential prognostic role of this target [41]. Therefore, VEDUM should currently be regarded as an investigational, yet promising, substrate-guided strategy.

## 8. Analysis of the Channels Displayed on Contrast-Enhanced Magnetic Resonance Imaging (MRI)

LGE-CMR can accurately detect normal ventricular tissue, dense scar, and scar border zone [42]. In parallel with the advances in functional left ventricular substrate mapping noted above, more detailed analysis of the tissue channels obtained by LGE-CMR has been carried out. It has been shown that specific channel characteristics determined by the special LGE-CMR software, such as the protected channel and its length, may be associated with the occurrence of ventricular arrhythmias [43, 44]. Therefore, identification of the CMR conduction channel (CMR-CC) may allow for pre-procedural planning. Reports have emerged that the functional arrhythmogenic substrate of scar-related VT and the CMR-CCs are spatially coincident. Vázquez-Calvo *et al*. [42] reported that in 42 patients with scar-related VT, 93.68% of the 95 ILAM-identified DZs were spatially consistent with the CMR-CCs, with 44.8% located in the mid-isthmus and 55.2% at the exit or entrance of the isthmus. Further, 32.5% of the DZs that appeared on re-mapping after ablation were consistent with the CMR-CCs, and their detection was used as an indicator for additional ablation targets. The combination of functional mapping and LGE-CMR has the potential to significantly improve the representation of functional substrates with high specificity. The relation between the CMR-CCs identified by the ADAS software system, ILAMs, and the estimated VT isthmus identified by the pace mapping method is shown in Fig. 4.

Cardiac CT offers an alternative to CMR when gadolinium administration or scanner availability is limited. CT-derived attenuation and wall-thickness maps—registered to the electroanatomic map (EAM)—can delineate putative conduction channels and anchor sinus-rhythm functional mapping cues [45]. However, variability in acquisition and segmentation persists, underscoring the need for prospective standardization.

To aid synthesis, Table 1 provides a side-by-side comparison of functional mapping strategies—ILAM, DEEP/Sense, near-field/peak-frequency mapping, intramural delay mapping, imaging-guided mapping (CMR/CT), scar dechanneling, and VEDUM—summarizing their physiologic premise, strengths, limitations, and current level of clinical evidence.

**Table 1.** **Comparative overview of functional substrate–mapping strategies for scar-related VT**.

Strategy	Physiologic premise/protocol cue	Pros	Cons/caveats	Level of clinical evidence
ILAM (isochronal late activation mapping)	Sinus-rhythm activation annotated into isochrones; deceleration zones identified by isochronal crowding (DZ)	Localizes reentry-vulnerable zones; targetable under sinus rhythm; supported by multicenter data	Annotation dependence; threshold sensitivity; may miss mid-myocardial circuits; operator training required	Validated (multicenter)
DEEP/Sense-protocol extrastimulus mapping	Short-coupled extrastimuli under SR to unmask decrement-evoked potentials and lines of block; Sense uses single extra beats	Functionally probes channels without sustained VT; improves specificity near circuit boundary; favorable multicenter outcomes	Protocol variability (site/output/coupling); annotation dependence; hemodynamic burden varies; standardization needed	Supported (multicenter)
Near-field/Peak-frequency (PF) mapping	Automated near-field detection and peak-frequency metric to identify local activation and high-PF zones in low-voltage areas	Rapid, more objective annotation; highlights targets; spatial concordance with DZs in early reports	Platform-specific; parameter/threshold sensitivity; prospective validation required	Emerging
Intramural delay mapping	High-density orthogonal sampling and endo–epi integration to infer in-wall slow conduction bridging otherwise discontinuous findings	Sensitive to concealed channels when surface voltage is nonspecific; bridges otherwise discontinuous endo–epi findings	Requires endo–epi access/integration; time-consuming acquisition; interpretation expertise needed	Emerging
Imaging-guided functional mapping (LGE-CMR/CT)	LGE-CMR conduction-channel analysis; CT attenuation and wall-thickness maps registered to EAM to delineate putative channels	Noninductive overview; aids access planning and corridor delineation; CT provides alternative when CMR is contraindicated/unavailable	Segmentation thresholds; motion/beam-hardening artefacts (CT); registration accuracy; availability constraints	Increasing adoption; supportive multicenter data
Scar dechanneling/LP-LAVA elimination	Target late potentials and LAVA channels within scar to interrupt conduction corridors	Intuitive endpoint; can reduce recurrence when combined with substrate modification	May require extensive ablation and longer procedures; potential complications; may not isolate functional isthmus	Established (legacy + contemporary)
VEDUM (ventricular electrogram-duration mapping)	VEDUM has two forms: a VT-rhythm version identifying diastolic long-duration EGMs, and a stable-rhythm version in sinus or paced rhythm without extrastimuli mapping slow-conduction corridors.	Provides functional cues without sustained VT (SR approach); hypothesis-generating multicenter experience	Heterogeneous implementations and positivity criteria; protocol standardization and external validation needed	Investigational/Early multicenter experience

This schematic summarizes physiologic premise/protocol cue, strengths, limitations, and level of clinical evidence across ILAM; DEEP/Sense-protocol extrastimulus mapping; near-field/peak-frequency (PF) mapping; intramural delay mapping; imaging-guided functional mapping (CMR/CT); scar dechanneling; and VEDUM. Abbreviations: ILAM, isochronal late activation mapping; DEEP, decrement-evoked potentials; PF, peak frequency; CMR, cardiovascular magnetic resonance; CT, computed tomography; DZ, deceleration zone; SR, sinus rhythm; EGM, electrogram; EAM, electroanatomic map; VT, ventricular tachycardia; LAVA, local abnormal ventricular activity; LP, late potential; LGE, late gadolinium enhancement; LOB, line of block.

## 9. Limitations and Adoption

Although clinically useful, these methods remain limited by annotation dependence, center-specific protocols, and inter-platform variability. Current adoption is concentrated in high-volume VT centers, underscoring the need for standardized workflows, operator training, and prospective multicenter validation with harmonized reporting.

## 10. Conclusions

Extensive substrate modification across the entire border zone and scar is time-consuming and may be impractical in patients with limited cardiac function or large substrates. Among sinus-rhythm functional strategies, ILAM and extra-stimulus–based mapping (DEEP/Sense) are supported by stronger clinical evidence and can be considered in current practice as adjuncts to substrate-based ablation. Integration with LGE-CMR conduction-channel analysis and multi-directional activation mapping shows increasing uptake with supportive multicenter data, whereas near-field/peak-frequency annotation approaches, omnipolar/near-field analyses, and CT-based functional inference should be regarded as emerging adjuncts. To establish clinical value and facilitate dissemination, standardized workflows, operator training, and prospective—ideally randomized—trials with harmonized reporting are needed.

## References

[1] Zeppenfeld K, Tfelt-Hansen J, de Riva M, Winkel BG, Behr ER, Blom NA (2022). 2022 ESC Guidelines for the management of patients with ventricular arrhythmias and the prevention of sudden cardiac death. *European Heart Journal*.

[2] Sapp JL, Parkash R, Tang ASL (2016). Ventricular Tachycardia Ablation versus Antiarrhythmic-Drug Escalation. *The New England Journal of Medicine*.

[3] Martinez BK, Baker WL, Konopka A, Giannelli D, Coleman CI, Kluger J (2020). Systematic review and meta-analysis of catheter ablation of ventricular tachycardia in ischemic heart disease. *Heart Rhythm*.

[4] Stevenson WG, Richardson TD, Kanagasundram AN, Tandri H (2024). State of the Art: Mapping Strategies to Guide Ablation in Ischemic Heart Disease. *JACC. Clinical Electrophysiology*.

[5] Tung R, Raiman M, Liao H, Zhan X, Chung FP, Nagel R (2020). Simultaneous Endocardial and Epicardial Delineation of 3D Reentrant Ventricular Tachycardia. *Journal of the American College of Cardiology*.

[6] Vlachos K, Letsas KP, Srinivasan NT, Frontera A, Efremidis M, Dragasis S (2022). The value of functional substrate mapping in ventricular tachycardia ablation. *Heart Rhythm O2*.

[7] Marchlinski FE, Callans DJ, Gottlieb CD, Zado E (2000). Linear ablation lesions for control of unmappable ventricular tachycardia in patients with ischemic and nonischemic cardiomyopathy. *Circulation*.

[8] Arenal A, Glez-Torrecilla E, Ortiz M, Villacastín J, Fdez-Portales J, Sousa E (2003). Ablation of electrograms with an isolated, delayed component as treatment of unmappable monomorphic ventricular tachycardias in patients with structural heart disease. *Journal of the American College of Cardiology*.

[9] Sacher F, Lim HS, Derval N, Denis A, Berte B, Yamashita S (2015). Substrate mapping and ablation for ventricular tachycardia: the LAVA approach. *Journal of Cardiovascular Electrophysiology*.

[10] Vergara P, Trevisi N, Ricco A, Petracca F, Baratto F, Cireddu M (2012). Late potentials abolition as an additional technique for reduction of arrhythmia recurrence in scar related ventricular tachycardia ablation. *Journal of Cardiovascular Electrophysiology*.

[11] Di Biase L, Burkhardt JD, Lakkireddy D, Carbucicchio C, Mohanty S, Mohanty P (2015). Ablation of Stable VTs Versus Substrate Ablation in Ischemic Cardiomyopathy: The VISTA Randomized Multicenter Trial. *Journal of the American College of Cardiology*.

[12] Tung R, Vaseghi M, Frankel DS, Vergara P, Di Biase L, Nagashima K (2015). Freedom from recurrent ventricular tachycardia after catheter ablation is associated with improved survival in patients with structural heart disease: An International VT Ablation Center Collaborative Group study. *Heart Rhythm*.

[13] Anter E, Tschabrunn CM, Buxton AE, Josephson ME (2016). High-Resolution Mapping of Postinfarction Reentrant Ventricular Tachycardia: Electrophysiological Characterization of the Circuit. *Circulation*.

[14] Berte B, Relan J, Sacher F, Pillois X, Appetiti A, Yamashita S (2015). Impact of Electrode Type on Mapping of Scar-Related VT. *Journal of Cardiovascular Electrophysiology*.

[15] Martin CA, Takigawa M, Martin R, Maury P, Meyer C, Wong T (2019). Use of Novel Electrogram “Lumipoint” Algorithm to Detect Critical Isthmus and Abnormal Potentials for Ablation in Ventricular Tachycardia. *JACC. Clinical Electrophysiology*.

[16] Okubo K, Frontera A, Bisceglia C, Paglino G, Radinovic A, Foppoli L (2019). Grid Mapping Catheter for Ventricular Tachycardia Ablation. *Circulation. Arrhythmia and Electrophysiology*.

[17] Hawson J, Anderson RD, Das SK, Al-Kaisey A, Chieng D, Segan L (2024). Optimal Annotation of Local Activation Time in Ventricular Tachycardia Substrate Mapping. *JACC. Clinical Electrophysiology*.

[18] Payne JE, Woods C, Elshazly MB, Matthews A, Kroman A, Feng Z (2024). A novel automated peak frequency annotation algorithm for identifying deceleration zones and ventricular tachycardia ablation sites. *Heart Rhythm*.

[19] Aziz Z, Shatz D, Raiman M, Upadhyay GA, Beaser AD, Besser SA (2019). Targeted Ablation of Ventricular Tachycardia Guided by Wavefront Discontinuities During Sinus Rhythm: A New Functional Substrate Mapping Strategy. *Circulation*.

[20] Anter E, Neuzil P, Reddy VY, Petru J, Park KM, Sroubek J (2020). Ablation of Reentry-Vulnerable Zones Determined by Left Ventricular Activation From Multiple Directions: A Novel Approach for Ventricular Tachycardia Ablation: A Multicenter Study (PHYSIO-VT). *Circulation. Arrhythmia and Electrophysiology*.

[21] Khan H, Bonvissuto MR, Rosinski E, Shokr M, Metcalf K, Jankelson L (2023). Comparison of combined substrate-based mapping techniques to identify critical sites for ventricular tachycardia ablation. *Heart Rhythm*.

[22] Tonko JB, Lozano C, Moreno J, Chow A, Dhinoja M, Lambiase PD (2024). Near-field detection and peak frequency metric for substrate and activation mapping of ventricular tachycardias in two- and three-dimensional circuits. *Europace: European Pacing, Arrhythmias, and Cardiac Electrophysiology: Journal of the Working Groups on Cardiac Pacing, Arrhythmias, and Cardiac Cellular Electrophysiology of the European Society of Cardiology*.

[23] Irie T, Yu R, Bradfield JS, Vaseghi M, Buch EF, Ajijola O (2015). Relationship between sinus rhythm late activation zones and critical sites for scar-related ventricular tachycardia: systematic analysis of isochronal late activation mapping. *Circulation. Arrhythmia and Electrophysiology*.

[24] Aziz Z, Tung R (2018). Novel Mapping Strategies for Ventricular Tachycardia Ablation. *Current Treatment Options in Cardiovascular Medicine*.

[25] Anter E, Kleber AG, Rottmann M, Leshem E, Barkagan M, Tschabrunn CM (2018). Infarct-Related Ventricular Tachycardia: Redefining the Electrophysiological Substrate of the Isthmus During Sinus Rhythm. *JACC. Clinical Electrophysiology*.

[26] Brunckhorst CB, Delacretaz E, Soejima K, Maisel WH, Friedman PL, Stevenson WG (2004). Identification of the ventricular tachycardia isthmus after infarction by pace mapping. *Circulation*.

[27] de Chillou C, Groben L, Magnin-Poull I, Andronache M, MagdiAbbas M, Zhang N (2014). Localizing the critical isthmus of postinfarct ventricular tachycardia: the value of pace-mapping during sinus rhythm. *Heart Rhythm*.

[28] Tung R, Josephson ME, Bradfield JS, Shivkumar K (2016). Directional Influences of Ventricular Activation on Myocardial Scar Characterization: Voltage Mapping With Multiple Wavefronts During Ventricular Tachycardia Ablation. *Circulation. Arrhythmia and Electrophysiology*.

[29] Martin CA, Martin R, Maury P, Meyer C, Wong T, Dallet C (2019). Effect of Activation Wavefront on Electrogram Characteristics During Ventricular Tachycardia Ablation. *Circulation. Arrhythmia and Electrophysiology*.

[30] Kuroki K, Nogami A, Igarashi M, Masuda K, Kowase S, Kurosaki K (2018). New Substrate-Guided Method of Predicting Slow Conducting Isthmuses of Ventricular Tachycardia: Preliminary Analysis to the Combined Use of Voltage Limit Adjustment and Fast-Fourier Transform Analysis. *Circulation. Arrhythmia and Electrophysiology*.

[31] Bhaskaran A, Fitzgerald J, Jackson N, Gizurarson S, Nanthakumar K, Porta-Sánchez A (2020). Decrement Evoked Potential Mapping to Guide Ventricular Tachycardia Ablation: Elucidating the Functional Substrate. *Arrhythmia & Electrophysiology Review*.

[32] Acosta J, Andreu D, Penela D, Cabrera M, Carlosena A, Korshunov V (2018). Elucidation of hidden slow conduction by double ventricular extrastimuli: a method for further arrhythmic substrate identification in ventricular tachycardia ablation procedures. *Europace: European Pacing, Arrhythmias, and Cardiac Electrophysiology: Journal of the Working Groups on Cardiac Pacing, Arrhythmias, and Cardiac Cellular Electrophysiology of the European Society of Cardiology*.

[33] Jaïs P, Maury P, Khairy P, Sacher F, Nault I, Komatsu Y (2012). Elimination of local abnormal ventricular activities: a new end point for substrate modification in patients with scar-related ventricular tachycardia. *Circulation*.

[34] Jackson N, Gizurarson S, Viswanathan K, King B, Massé S, Kusha M (2015). Decrement Evoked Potential Mapping: Basis of a Mechanistic Strategy for Ventricular Tachycardia Ablation. *Circulation. Arrhythmia and Electrophysiology*.

[35] Porta-Sánchez A, Jackson N, Lukac P, Kristiansen SB, Nielsen JM, Gizurarson S (2018). Multicenter Study of Ischemic Ventricular Tachycardia Ablation With Decrement-Evoked Potential (DEEP) Mapping With Extra Stimulus. *JACC. Clinical Electrophysiology*.

[36] Srinivasan NT, Garcia J, Schilling RJ, Ahsan S, Babu GG, Ang R (2020). Multicenter Study of Dynamic High-Density Functional Substrate Mapping Improves Identification of Substrate Targets for Ischemic Ventricular Tachycardia Ablation. *JACC. Clinical Electrophysiology*.

[37] Guichard JB, Regany-Closa M, Vázquez-Calvo S, Zazu B, Pellicer Sendra B, Serrano-Campaner J (2024). Substrate Mapping for Ventricular Tachycardia Ablation Through High-Density Whole-Chamber Double Extra Stimuli: The S3 Protocol. *JACC. Clinical Electrophysiology*.

[38] Hanson M, Enriquez A, Garcia F (2024). Intramural Ventricular Arrhythmias: How to Crack a Hard Nut. *Current Cardiology Reports*.

[39] Rossi P, Cauti FM, Niscola M, Calore F, Fanti V, Polselli M (2021). A novel Ventricular map of Electrograms DUration as a Method to identify areas of slow conduction for ventricular tachycardia ablation: The VEDUM pilot study. *Heart Rhythm*.

[40] Rossi P, Cauti FM, Niscola M, Magnocavallo M, Polselli M, Capone S (2023). Ventricular Electrograms Duration Map to Detect Ventricular Arrhythmia Substrate: the VEDUM Project Study. *Circulation. Arrhythmia and Electrophysiology*.

[41] Cauti FM, Rossi P, Magnocavallo M, Polselli M, Martini N, Fioravanti F (2025). Ventricular Duration Map Area as a Valuable and Effective Target for VT Ablation End Point in Ischemic Cardiomyopathy: The VEDUM FREEDOM Study. *Circulation. Arrhythmia and Electrophysiology*.

[42] Vázquez-Calvo S, Casanovas JM, Garre P, Ferró E, Sánchez-Somonte P, Quinto L (2023). Evolution of Deceleration Zones During Ventricular Tachycardia Ablation and Relation With Cardiac Magnetic Resonance. *JACC. Clinical Electrophysiology*.

[43] Sanchez-Somonte P, Garre P, Vázquez-Calvo S, Quinto L, Borràs R, Prat S (2023). Scar conducting channel characterization to predict arrhythmogenicity during ventricular tachycardia ablation. *Europace: European Pacing, Arrhythmias, and Cardiac Electrophysiology: Journal of the Working Groups on Cardiac Pacing, Arrhythmias, and Cardiac Cellular Electrophysiology of the European Society of Cardiology*.

[44] Vázquez-Calvo S, Mas Casanovas J, Garre P, Sánchez-Somonte P, Falzone PV, Uribe L (2024). Non-invasive detection of slow conduction with cardiac magnetic resonance imaging for ventricular tachycardia ablation. *Europace: European Pacing, Arrhythmias, and Cardiac Electrophysiology: Journal of the Working Groups on Cardiac Pacing, Arrhythmias, and Cardiac Cellular Electrophysiology of the European Society of Cardiology*.

[45] Laan D, Hopman LHGA, Figueras I Ventura RM, Soré B, Allaart CP, Kemme MJB (2025). Ventricular tachycardia substrate mapping with cardiac computed tomography and cardiac magnetic resonance imaging: Head-to-head comparison of two clinically available postprocessing platforms. *Heart Rhythm*.

